# Fluvoxamine monotherapy for psychotic depression: the potential role of sigma-1 receptors

**DOI:** 10.1186/1744-859X-8-26

**Published:** 2009-12-21

**Authors:** Tsutomu Furuse, Kenji Hashimoto

**Affiliations:** 1 Department of Psychiatry, Asahikawa Red Cross Hospital, Asahikawa, Japan; 2 Division of Clinical Neuroscience, Chiba University Center for Forensic Mental Health, Chiba, Japan

## Abstract

**Background:**

Psychotic depression is a clinical subtype of major depressive disorder. A number of clinical studies have demonstrated the efficacy of the combination of an antidepressant (for example, a tricyclic antidepressant or selective serotonin reuptake inhibitor (SSRI)) and an atypical antipsychotic or electroconvulsive therapy in treating psychotic depression. In some cases, the clinician or patient may prefer to avoid antipsychotic drugs altogether because of the risk of extrapyramidal side effects (EPS) in patients with psychotic depression treated with these drugs.

**Methods:**

We report five cases where fluvoxamine monotherapy was effective in the patients with psychotic depression.

**Results:**

The scores on the Hamilton Depression (HAM-D) scale and the Brief Psychiatric Rating Scale (BPRS) in the five patients with psychotic depression were reduced after fluvoxamine monotherapy.

**Conclusion:**

Doctors should consider fluvoxamine monotherapy as an alternative approach in treating psychotic depression because it avoids the risk of EPS from antipsychotic drugs.

## Background

Psychotic depression is a clinical subtype of major depressive disorder and is characterized by psychosis accompanied by relatively severe depressive symptoms that include psychomotor impairment, morbid cognition, suicidal ideation and neuropsychological impairment. Unfortunately, psychotic depression frequently proves difficult to treat. A number of clinical studies have demonstrated the efficacy of the combination of an antidepressant (for example, a tricyclic antidepressant or selective serotonin reuptake inhibitor (SSRI)) and an atypical antipsychotic or electroconvulsive therapy (ECT) in treating psychotic depression. In some cases, the clinician or patient may prefer to avoid antipsychotic drugs altogether because of the risk of extrapyramidal side effects (EPS) in patients with psychotic depression treated with these drugs [1].

Interestingly, monotherapy using the SSRI fluvoxamine was effective against both the psychotic and depressive symptoms of this disorder [2,3], whereas paroxetine had a lesser effect [4]. The reason underlying the difference in efficacy for these two SSRIs is currently unknown. Several pieces of evidence suggest that the endoplasmic reticulum protein sigma-1 receptors play a role in the pathophysiology of depression and in the active mechanisms of antidepressants [5,6]. Unlike paroxetine, with an inhibition constant (Ki) of 1893 nM, fluvoxamine is a potent sigma-1 receptor agonist with a Ki of 36 nM [5]. A positron emission tomography study demonstrated that fluvoxamine (50 to 200 mg), but not paroxetine (20 mg), binds to sigma-1 receptors in the intact human brain at therapeutic doses [5], suggesting that sigma-1 receptors are involved in the active mechanisms of fluvoxamine [5]. Based on all these findings, a hypothesis has been proposed that the sigma-1 receptors may be implicated in the efficacy of fluvoxamine for psychotic depression [7-9]. Here, we report five cases in which fluvoxamine monotherapy was effective in Japanese patients with psychotic depression.

## Case reports

Table 1 shows the characteristics of five patients with psychotic major depression diagnosed by Diagnostic and Statistical Manual of Mental Disorders, 4th Edition (DSM-IV) criteria. Case 1 was admitted to the hospital due to sleep disturbance and delusion. The Hamilton Depression (HAM-D) scale and the Brief Psychiatric Rating Scale (BPRS) scores were 34 and 66, respectively. Fluvoxamine (50 mg twice a day) and flunitrazepam (2 mg) were initiated, and the next day fluvoxamine was increased to 100 mg since there were no gastrointestinal side effects. The patient's condition was better 7 days after beginning treatment with fluvoxamine, but she still showed a tendency to want to go to bed. Therefore, fluvoxamine was increased to 150 mg. By 2 weeks later, her activity levels had recovered. Her HAM-D and BPRS scores had dramatically decreased (Table 1), and she was discharged home 3 weeks after beginning treatment with fluvoxamine.

**Table 1:** Characteristics and depression rating scores of patients with psychotic depression who responded to fluvoxamine monotherapy

					Pretreatment score		Post-treatment score	
					
Patient	Gender (F/M)	Age, years	Age at onset, years	Duration of illness	HAM-D	BPRS	HAM-D	BPRS
Case 1	F	72	72	6 weeks	34	66	7 (3 weeks)	22 (3 weeks)
Case 2	F	54	54	10 weeks	42	77	9 (6 weeks)	32 (6 weeks)
Case 3	M	67	67	6 weeks	45	73	5 (17 days)	26 (17 days)
Case 4	F	60	60	7 weeks	35	69	6 (3 weeks)	22 (3 weeks)
Case 5	F	62	62	8 weeks	45	80	5 (4 weeks)	22 (4 weeks)

BPRS = Brief Psychotic Rating Scale; HAM-D = Hamilton Depression Rating Scale.

Case 2 had delusions of being observed, and was admitted to the emergency centre of the hospital after eating cigarettes as a suicide attempt. The HAM-D and BPRS scores were 42 and 77, respectively. Fluvoxamine (50 mg twice a day) and flunitrazepam (2 mg) were initiated, and the next day increased to 100 mg. Fluvoxamine was further increased to 150 mg because she still had delusions 2 weeks after beginning treatment. Her HAM-D and BPRS scores decreased (Table 1), and she was discharged to her home 6 weeks after beginning treatment with fluvoxamine.

Case 3 requested an operation on a cataract, but was rejected because of the progression of cornea thinning. He was admitted to the emergency centre of the hospital due to paranoia and delusions. The HAM-D and BPRS scores were 45 and 73, respectively. Fluvoxamine (50 mg twice a day), flunitrazepam (2 mg), and etizolam (0.5 mg) were initiated, and the next day, fluvoxamine was increased to 100 mg. His HAM-D and BPRS scores dramatically decreased (Table 1), and he was discharged home 17 days after beginning treatment with fluvoxamine.

Case 4 had experienced paranoid symptoms for a week. The HAM-D and BPRS scores were 35 and 69, respectively. Fluvoxamine (50 mg twice a day) and flunitrazepam (2 mg) were initiated, and the next day, fluvoxamine was increased to 100 mg. She was better 2 weeks after the beginning of treatment with fluvoxamine. Her HAM-D and BPRS scores dramatically decreased (Table 1), and she was discharged home 3 weeks after beginning treatment with fluvoxamine.

Case 5 had paranoia and delusion. The HAM-D and BPRS scores were 45 and 80, respectively. Fluvoxamine (50 mg twice a day) and flunitrazepam (2 mg) were initiated; fluvoxamine was increased to 100 mg the next day, and to 150 mg 7 days later. By 3 weeks later, her paranoia and delusion had disappeared. Her HAM-D and BPRS scores had decreased (Table 1) and she was discharged home 4 weeks after the beginning of treatment with fluvoxamine.

## Discussion

Here we report that fluvoxamine was effective in five patients with psychotic depression. However, further studies using large numbers of patients are needed before it can be concluded that fluvoxamine monotherapy is effective in patients with psychotic depression. It seems that serotonin reuptake inhibition as well as sigma-1 receptor agonism may be involved in the active mechanism of fluvoxamine, since paroxetine had a lesser effect in psychotic depression [4]. Nonetheless, this study did not clarify whether sigma-1 receptors are involved in the active mechanism of fluvoxamine. To confirm the role of sigma-1 receptor agonism in the treatment of psychotic depression, a randomized double-blind study of fluvoxamine (a sigma-1 receptor agonist) and sertraline (a sigma-1 receptor antagonist)[5,6] in patients with psychotic depression might be helpful.

## Conclusions

These cases suggest that fluvoxamine could be an alternative approach in treating psychotic depression because of the risk of EPS by antipsychotic drugs. More detailed double-blind studies should be performed to clarify the role of sigma-1 receptors in the efficacy of fluvoxamine for psychotic depression.

## Consent

Written informed consents were obtained from the all patients in this case report.

## Competing interests

The authors declare that they have no competing interests.

## Authors' contributions

TF contributed to the clinical and rating evaluations during the follow-up periods. KH conceived of the study and participated in its study and coordination. Both authors read and approved the final manuscript.
